# Suicidal tendencies in Schizophrenia patients

**DOI:** 10.1192/j.eurpsy.2022.2029

**Published:** 2022-09-01

**Authors:** A. Pascariu, A. Bosun, R. Kalinovic, G. Vlad, V. Enatescu

**Affiliations:** 1 “Pius Brinzeu” Emergency County Hospital, Psychiatry Clinic I, Timisoara, Romania; 2 “Victor Babes” University of Medicine and Pharmacy, Genetics, Timisoara, Romania; 3 “Victor Babes” University of Medicine and Pharmacy, Biochemistry, TIMISOARA, Romania; 4 Victor Babes University of Medicine and Pharmacy Timisoara-Discipline of Psychiatry, Timisoara, Romania and Eduard Pamfil Psychiatry Clinic, Timisoara County Hospital, Psychiatry, Timisoara, Romania

**Keywords:** schizophrénia, Suicide, risk factors, PANSS

## Abstract

**Introduction:**

Suicide is one of the most frequent causes of death in schizophrenia patients, but the low insight in this pathology makes it difficult to identify persons at risk. The clinical picture of schizophrenia includes a wide variety of signs and symptoms, which make it hard to properly assess suicidal ideation.

**Objectives:**

Our study was aimed at comparing tendencies among the clinical presentation and sociodemographic factors of schizophrenic patients with and without suicide attempts in their medical history.

**Methods:**

We analysed the clinical data of 60 patients admitted to the Psychiatry Clinic of Timisoara with the diagnosis of Schizophrenia during 2020-2021. The PANNS-R scale was used for every patient, and each item was compared to the clinical data gathered.

**Results:**

A positive history of suicide attempts was corelated to blunted affect, stereotyped thinking, lack of spontanety and flow of conversation, somatic concern, tension, unsual tought content, lack of judgment and insight. Total negative symptoms were correlated with psychiatric family history.

**Conclusions:**

Considering a history of suicide attempts is a risk factor for suicide, more studies are needed to evaluate patients with such a history in order to identify the constelation of risk factors with a high predictibility value for suicide. This could help implement prophylactic measures in clinical practice that would decrease suicidal behaviour in schizophrenia.

**Disclosure:**

No significant relationships.

